# Enhancing patient education on the role of tibial osteotomy in the management of knee osteoarthritis using a customized ChatGPT: a readability and quality assessment

**DOI:** 10.3389/fdgth.2024.1480381

**Published:** 2025-01-03

**Authors:** Stephen Fahy, Stephan Oehme, Danko Dan Milinkovic, Benjamin Bartek

**Affiliations:** Centrum für Muskuloskeletale Chirurgie, Charité Universitätsmedizin Berlin, Berlin, Germany

**Keywords:** knee osteoarthritis, high tibial osteotomy, patient education, ChatGPT, readability, DISCERN criteria, artificial intelligence

## Abstract

**Introduction:**

Knee osteoarthritis (OA) significantly impacts the quality of life of those afflicted, with many patients eventually requiring surgical intervention. While Total Knee Arthroplasty (TKA) is common, it may not be suitable for younger patients with unicompartmental OA, who might benefit more from High Tibial Osteotomy (HTO). Effective patient education is crucial for informed decision-making, yet most online health information has been found to be too complex for the average patient to understand. AI tools like ChatGPT may offer a solution, but their outputs often exceed the public's literacy level. This study assessed whether a customised ChatGPT could be utilized to improve readability and source accuracy in patient education on Knee OA and tibial osteotomy.

**Methods:**

Commonly asked questions about HTO were gathered using Google's “People Also Asked” feature and formatted to an 8th-grade reading level. Two ChatGPT-4 models were compared: a native version and a fine-tuned model (“The Knee Guide”) optimized for readability and source citation through Instruction-Based Fine-Tuning (IBFT) and Reinforcement Learning from Human Feedback (RLHF). The responses were evaluated for quality using the DISCERN criteria and readability using the Flesch Reading Ease Score (FRES) and Flesch-Kincaid Grade Level (FKGL).

**Results:**

The native ChatGPT-4 model scored a mean DISCERN score of 38.41 (range 25–46), indicating poor quality, while “The Knee Guide” scored 45.9 (range 33–66), indicating moderate quality. Cronbach's Alpha was 0.86, indicating good interrater reliability. “The Knee Guide” achieved better readability with a mean FKGL of 8.2 (range 5–10.7, ±1.42) and a mean FRES of 60 (range 47–76, ±7.83), compared to the native model's FKGL of 13.9 (range 11–16, ±1.39) and FRES of 32 (range 14–47, ±8.3). These differences were statistically significant (*p* < 0.001).

**Conclusions:**

Fine-tuning ChatGPT significantly improved the readability and quality of HTO-related information. “The Knee Guide” demonstrated the potential of customized AI tools in enhancing patient education by making complex medical information more accessible and understandable.

## Introduction

Osteoarthritis (OA) of the Knee is a common and debilitating condition, with an estimated lifetime risk of 40% among men and 47% among women (1). The disease causes a significant impairment in physical function which in turn can have a significant impact on the mental health of those afflicted (2, 3). Traditionally, in the early stages of the disease non-surgical treatment modalities such as physiotherapy, bracing, and joint injections are employed to relieve symptoms and to try and prevent further degenerative changes. The efficacy of non-operative management often wains with disease progression, leading patients to explore more invasive surgical options, namely Total Knee Arthroplasty (TKA), Unicompartmental Knee Arthroplasty (UKA), and corrective osteotomy. While TKA is an extremely effective procedure, it is not the right procedure for every patient. Approximately 20% of patients report dissatisfaction post-operatively, typically younger patients with higher functional demands and as such, higher postoperative expectations (4). For patients with symptomatic unicompartmental disease, procedures such as UKA and High Tibial Osteotomy (HTO) are well-established treatment options.

Owing to the multitude of treatment options available, careful patient selection is paramount. Preoperative patient education is essential to ensure patients have a good understanding of the indications, risks, and potential benefits of the surgical options available to them, as well as realistic expectations regarding postoperative function. The ability of patients to obtain, interpret and use medical information is referred to as “health literacy” (5). Historically, pre-operative education was delivered directly from healthcare providers to patients through in-patient consultation, or via Patient Education Materials (PEMS). Since the early 1990s, the widespread rollout of the Internet has transformed how patients access health information. Patients view the Internet as a valuable resource for education, often reporting that internet-based resources are equivalent to, or superior than, information received from healthcare providers (6, 7). Despite this, both traditional PEMS, as well as Internet-based resources have been consistently shown to be written at literacy levels far exceeding the average level of literacy of the general public (8–10). This has also been observed in internet-based resources on osteotomies around the knee joint (11). Various studies have shown, that the average literacy level in America is in keeping with that of an 8th grade reading level (12–15). As such, it is pivotal that health information be delivered at or below this level to maximize retention, optimize health literacy, and ultimately facilitate the development of realistic post-operative expectations. Artificial Intelligence (AI) tools such as ChatGPT, have recently gained popularity among the general public. These tools have the potential to replace both traditional PEMS and Internet-based resources by providing patients with rapid access to individualised information about their conditions and treatment options. Previous studies in the field of orthopaedics have found that information provided by Large Language Models (LLMs) like ChatGPT is often of moderate to good quality, however, the content has been consistently found to be too complex for the general public (16–18). Recent iterations of ChatGPT allow for the development of custom ChatGPTs tailored to specific tasks. We hypothesized that when asked common patient-related queries about high tibial osteotomies, a customised ChatGPT would produce information of similar quality to the ChatGPT 4 with significantly improved readability, ultimately making it more useful as a tool for patient education.

## Methods

### Question generation

On 05.05.2024, the terms “High Tibial Osteotomy”, “HTO”, and “Knee Osteotomy” were entered into a Google (www.google.com) Internet browser. Google generates a “People Also Asked” (PAA) section for topics searched, listing the most commonly asked questions by users relating to a given search term. The results page was refreshed until the top 30 questions were generated for each search term, duplicate questions were removed leaving 25 questions for final analysis. This technique is commonly employed in studies assessing the information-seeking behaviour of patients (19–21). A freshly installed browser and Virtual Private Network (VPN) was utilized to limit the influence of previous search terms on our results. The questions were subsequently formatted to ensure that they were written at, or below, the average American reading level of 8th grade so as to not artificially inflate the complexity of the responses given by ChatGPT (see Table 1).

**Table 1:** HTO question list.

1.	What is a HTO?
2.	What is the purpose of a HTO?
3.	How does a HTO work?
4.	What happens during HTO surgery?
5.	What type of bone graft is used in a HTO?
6.	Where is HTO surgery performed?
7.	How does HTO differ from knee replacement surgery?
8.	What other procedures can be done instead of a knee replacement?
9.	How successful is a HTO?
10.	Can a HTO fail? What is the failure rate?
11.	How painful is a HTO?
12.	How long does recovery take after a HTO?
13.	What should I expect directly after my HTO?
14.	How do I sleep after a HTO?
15.	Can I walk straight away after a HTO?
16.	How long will I need crutches after a HTO?
17.	What is the aftercare required following a HTO?
18.	Do I need a cast after a HTO?
19.	How long before I can return to work after a HTO?
20.	When can I drive after a HTO?
21.	When can I return to sports after a HTO?
22.	How long will I stay in the hospital after a HTO?
23.	At what age is a HTO most suitable?
24.	What are the risks and draw backs of a HTO over a knee replacement?
25.	What are the benefits and advantages of a HTO over a knee replacement?
Flesch-Kincaid Reading Grade Level 3.0	
Gunning Fox Index: 6.5	
Flesch Reading Ease 87.6	

### ChatGPT-4 configuration

#### Native ChatGPT-4

The native ChatGPT-4 model used in this study was the standard version provided by OpenAI, without any additional fine-tuning or customization. This model represents a general-purpose configuration designed to generate responses across a broad spectrum of topics using the architecture of GPT-4.

#### Fine-tuned ChatGPT-4 “knee guide”

The custom ChatGPT model, named “The Knee Guide,” was fine-tuned to ensure it placed particular emphasis on readability, clarity, and the accuracy of source citation in the responses it produced. This was performed using two main techniques:

Instruction-Based Fine-Tuning: The model was fine-tuned using a set of instructions to produce responses at a 8th-grade reading level. This involved instructing the model to use simple, direct language and avoid words with three or more syllables. It was instructed to refrain from using medical jargon and to replace it with commonly understood terms. For example, the term “osteotomy” was referred to as “HTO” with a simple definition provided.

Reinforcement Learning from Human Feedback (RLHF): This technique allowed refinement of the model through the provision of feedback on its responses. This feedback loop helped improve the clarity, readability, and accuracy of the information provided by the model. Human reviewers assessed the model's outputs and guided it towards producing more user-friendly and well-sourced responses before its utilisation for the topic of HTO.

### Data collection and analysis

On 07.05.2024 the questions were posed to both the native ChatGPT-4 and the custom Knee Guide model simultaneously. Each response was saved in a separate Microsoft Word document for ease of analysis. Hyperlinks were removed from the responses to ensure the readability software focused solely on textual readability.

### Quality assessment

The DISCERN criteria were used to assess the quality of the responses given. The DISCERN criteria are frequently used for the assessment of the quality of written consumer health information, either online or in PEMs. It consists of 16 questions, each rated from 1 to 5. Questions 1–8 assess content reliability, and questions 9–15 directly assess the information provided regarding treatment choices including the potential benefits, risks, and alternative treatments available. The final question assesses the perceived quality of all of the information provided. The maximum score is 80, with scores of 70 and above deemed “excellent”, and scores of 50 and above deemed “good” (22). Three experienced orthopaedic surgeons specialized in the field of Knee surgery (listed authors (BB, SO, DM), rated the responses yielded by the two models. The raters were blinded to the ChatGPT model used.

### Assessment of readability

The Readability Studio Professional Edition Program (Oleander Software Ltd., version 2019) was used for the assessment of readability (23). This software evaluates readability using a host of assessment tools to assess the complexity of a text, in this study we assessed readability using the Flesch Reading Ease Score (FRES) and the Flesch–Kincaid Reading Grade Level (FKGL) (Supplementary Table 1). The Reading Grade Levels (RGLs) reported are indicative of the United States (US) grade level required to comprehend the text. The FRES rates the complexity of a text as a score from 0 to 100, with scores of 60 and above recognized as “plain English”, which would be easily understood by 13- to 15-year-old students.

### Statistical analysis

Statistical analysis was performed using IBM SPSS Statistics (version 29.0.0.0). Interrater reliability for DISCERN Scores was evaluated using the intraclass correlation coefficient within a two-way mixed model. To determine statistically significant differences between groups, the Wilcoxon matched-pairs signed rank test was employed, focusing on the mean total DISCERN criteria score, the mean score per DISCERN criteria category, and readability. This study required no ethical approval.

## Results

### DISCERN score

The mean DISCERN score of responses given by ChatGPT 4 was 38.41 (range 25–46), with the maximum score being 80, indicating responses of poor quality. The mean DISCERN score for answers given by “the Knee Guide” was 45.95 (range 33–66), indicating responses of moderate quality. Cronbach's Alpha was 0.86, indicating good interrater reliability. “The Knee Guide” had a significantly higher DISCERN score than ChatGPT 4 (*p* < 0.001). “The Knee Guide” was significantly better than ChatGPT4 concerning the clarity of the responses yielded, response relevance, source citation, the provision of external sources, discussion of potential treatment benefits, stressing the availability of alternative treatments, the consequences of conservative management, as well as the importance of shared decision making between patient and healthcare professional.

### Readability

**Figure 1:**
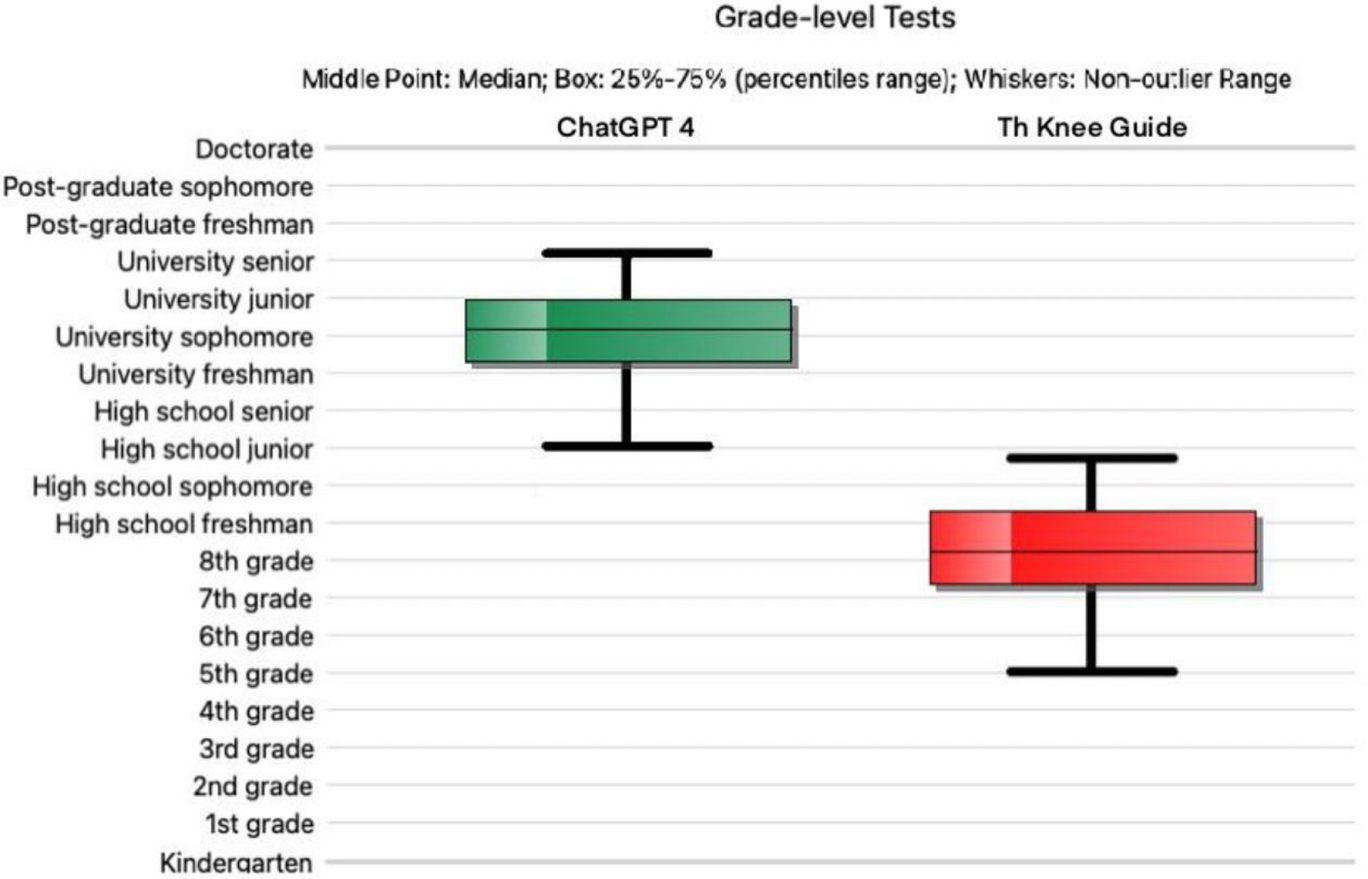
Reading grade level for ChatGPT version 4 (green) vs the “fine-tuned” ChatGPT (red). The horizontal line denotes the median; the upper and lower bounds of each box depict the interquartile range; the whiskers show the lower and upper quartiles.

Answers produced by “The Knee Guide” had a significantly lower RGL than those produced by ChatGPT 4 (*p* ≤ 0.001). The mean FKGL for the “Knee Guide” was 8.2 (range 5–10.7, ±1.42), while responses given by ChatGPT 4 had a mean FKGL of 13.99 (range 11–16, ±1.39) (Figure 1). Of the answers given by ChatGPT 4, none were written at or below the recommended 8th grade reading level. Furthermore, the FRES was significantly higher in the responses given by “The Knee Guide” in comparison with ChatGPT4. The mean FRES of “The Knee Guide” was 60 (range 47–76, ±7.83) indicating good readability consistent with an 8th grade reading level, while ChatGPT 4 had a mean FRES of 32 (range 14–47, ±8.3) indicating significant complexity in the level of readability of responses produced consistent with an RGL of a College graduate. A significant between-groups difference was observed (*p* ≤ 0.001).

## Discussion

Our research aimed to assess whether a fine-tuned ChatGPT could provide patients with good quality information in relation to tibial osteotomies and Knee osteoarthritis at an appropriate RGL for the general public. The corresponding author was responsible for fine-tuning the custom ChatGPT model, this was achieved through both Instruction-Based Fine-Tuning (IBFT) as well as Reinforcement Learning from Human Feedback (RLHF). Both of these techniques are commonly employed to fine-tune LLMs, and owing to their intuitive nature, are time-efficient and require little prior knowledge or skill to implement effectively.

ChatGPT has been previously found to provide moderate to good quality information for a host of orthopaedic conditions, including ACL reconstruction, Shoulder stabilisation surgery and Knee osteoarthritis (16–18). Our research found that ChatGPT 4 produced responses deemed to be of poor quality when posed with common patient questions on high tibial osteotomies. ChatGPT 4 consistently scored poorly with respect to source citation and provision of external supports for patients. In contrast, the “fine-tuned” ChatGPT was deemed to produce answers of fair to good quality. The “Knee-Guide” was significantly better ChatGPT 4 in almost every DISCERN category. The fine tuning process allowed the model to provide clear, understandable, and personalised information to patients, as well as placing a particular emphasis on clarity of source citation and provision of external sources, both key factors in improving the trustworthiness of the information produced for patients and healthcare providers.

Our research highlighted a consistent problem with AI tools like ChatGPT, in that the complexity of responses given often far exceeds the literacy levels of the general public. The mean reading grade level of responses given by ChatGPT 4 was 13.99, consistent with a university sophomore, with no responses being delivered at the 8th grade reading level. The use of such complex language creates an unnecessary barrier to the widespread utilization of LLMs as a tool for patient education. Our research has demonstrated, that with little expertise, and minimal time commitment, LLMs like ChatGPT can be fine-tuned to drastically improve their accessibility, and as such make them valuable tools for patient education.

Our study is not without limitations, we chose to utilize the DISCERN score to assess the quality of information provided by ChatGPT. We chose the DISCERN score as it is the most commonly used scoring tool for the assessment of internet-based patient education material. While the DISCERN score has been shown to be both reliable and reproducible in the assessment of online PEMS and traditional patient education leaflets, its reliability in the assessment of LLMs is currently unknown. Owing to the conversational nature of LLMs it is likely that the assessment of isolated responses given by LLMs may yield artificially low scores. The development of a reliable and reproducible quality assessment tool for application in LLMs is an important topic for future research.

An additional limitation of the study is the realisation that ChatGPT is a continuously evolving and ever-improving tool. The quality assessment we conducted may quickly become outdated following the release of newer iterations. Significant advances have already been observed between ChatGPT-3.5 and GPT-4, with GPT-4 providing substantially more accurate and comprehensive patient education materials than ChatGPT 3.5, with an estimated improvement in response quality of approximately 30% (24, 25). This dynamic nature of LLMs underscores the need for continuous evaluation and adaptation in their applications.

## Conclusion

Our research evaluated the quality and readability of information produced by ChatGPT-4 and a fine-tuned ChatGPT program in relation to tibial osteotomies. 80% of patients are of the opinion that AI has the potential to improve healthcare quality, reduce costs, and increase accessibility and as such it is vital that its utility in the provision of healthcare information is scrutinized. We found that ChatGPT-4 provided poor-quality responses in relation to HTO, with insufficient source citation and a lack of external support provision. However, through the use of minimal “fine-tuning” ChatGPT can be utilized to deliver fair to good quality answers, significantly outperforming ChatGPT-4 with respect to response quality and readability. This was achieved through Instruction-Based Fine-Tuning (IBFT) and Reinforcement Learning from Human Feedback (RLHF), enabling the model to provide clear, personalized, and trustworthy information.

Another potential weakness in the use of AI tools like ChatGPT in patient education is the complexity of language produced in the responses given. ChatGPT-4's responses had a mean RGL of 13.99, far above the average American RGL of 8th-grade level. Again, we noted that through minimal “fine tuning” this obstacle can be overcome to enhance readability of responses produced by ChatGPT. In the future, further fine tuning of ChatGPT models may see them become reliable tools for patient education, delivering highly customized and reliable information content to patients in a time efficient manner.

## Data Availability

The raw data supporting the conclusions of this article will be made available by the authors, without undue reservation.

## References

[1] Johnson VL Hunter DJ. The epidemiology of osteoarthritis. Best Pract Res Clin Rheumatol. (2014) 28:5–15. 10.1016/j.berh.2014.01.004 24792942

[2] Guccione AA Felson DT Anderson JJ Anthony JM Zhang Y Wilson PW The effects of specific medical conditions on the functional limitations of elders in the Framingham study. Am J Public Health. (1994) 84:351–8. 10.2105/AJPH.84.3.351 8129049 PMC1614827

[3] Fahy S Moore J Kelly M Irwin S Kenny P. Assessing the attitudes, awareness, and behavioral alterations of patients awaiting total hip arthroplasty during the COVID-19 crisis. Geriatr Orthop Surg Rehabil. (2020) 11:215145932096937. 10.1177/2151459320969377 PMC7588755 33173605

[4] Bourne RB Chesworth BM Davis AM Mahomed NN Charron KDJ. Patient satisfaction after total knee arthroplasty: who is satisfied and who is not? Clin Orthop Relat Res. (2010) 468:57–63. 10.1007/s11999-009-1119-9 19844772 PMC2795819

[5] Wang C Li H Li L Xu D Kane RL Meng Q. Health literacy and ethnic disparities in health-related quality of life among rural women: results from a Chinese poor minority area. Health Qual Life Outcomes. (2013) 11:153. 10.1186/1477-7525-11-153 24020618 PMC3847672

[6] Diaz JA Griffith RA Ng JJ Reinert SE Friedmann PD Moulton AW. Patients’ use of the internet for medical information. J Gen Intern Med. (2002) 17:180–5. 10.1046/j.1525-1497.2002.10603.x 11929503 PMC1495021

[7] Hautala GS Comadoll SM Raffetto ML Ducas GW Jacobs CA Aneja A Most orthopaedic trauma patients are using the internet, but do you know where they’re going? Injury. (2021) 52:3299–303. 10.1016/j.injury.2021.02.029 33653619

[8] Doinn TÓ Broderick JM Abdelhalim MM Quinlan JF. Readability of patient educational materials in hip and knee arthroplasty: has a decade made a difference? J Arthroplasty. (2020) 35:3076–83. 10.1016/j.arth.2020.05.076 32631729

[9] Ó Doinn T Broderick JM Abdelhalim MM Quinlan JF. Readability of patient educational materials in pediatric orthopaedics. J Bone Joint Surg. (2021) 103:e47. 10.2106/JBJS.20.01347 33543881

[10] Ó Doinn T Broderick JM Clarke R Hogan N. Readability of patient educational materials in sports medicine. Orthop J Sports Med. (2022) 10:232596712210923. 10.1177/23259671221092356 PMC9082750 35547607

[11] Broderick JM McCarthy A Hogan N. Osteotomy around the knee: assessment of quality, content and readability of online information. Knee. (2021) 28:139–50. 10.1016/j.knee.2020.11.010 33360380

[12] Kirsch I Jungeblut A Jenkins L Kolstad A. Adult Literacy in America: A First Look at the Results of the National Adult Literacy Survey. Washington DC: U.S. Department of Education, National Center for Education Statistics (1993).

[13] Weis BD. Health Literacy: A Manual for Clinicians. Chicago: American Medical Association, American Medical Foundation (2003).

[14] Cotugna N Vickery CE Carpenter-Haefele KM. Evaluation of literacy level of patient education pages in health-related journals. J Community Health. (2005) 30:213–9. 10.1007/s10900-004-1959-x 15847246

[15] Brega AG Freedman MAG LeBlanc WG Barnard J Mabachi NM Cifuentes M Using the health literacy universal precautions toolkit to improve the quality of patient materials. J Health Commun. (2015) 20:69–76. 10.1080/10810730.2015.1081997 26513033 PMC5085259

[16] Hurley ET Crook BS Lorentz SG Danilkowicz RM Lau BC Taylor DC Evaluation high-quality of information from ChatGPT (artificial intelligence—large language model) artificial intelligence on shoulder stabilization surgery. Arthroscopy. (2023) 39:25–28. 10.1016/j.arthro.2023.07.048 37567487

[17] Fahy S Niemann M Böhm P Winkler T Oehme S. Assessment of the quality and readability of information provided by ChatGPT in relation to the use of platelet-rich plasma therapy for osteoarthritis. J Pers Med. (2024a) 14:495. 10.3390/jpm14050495 38793077 PMC11122161

[18] Fahy S Oehme S Milinkovic D Jung T Bartek B. Assessment of quality and readability of information provided by ChatGPT in relation to anterior cruciate ligament injury. J Pers Med. (2024b) 14:104. 10.3390/jpm14010104 38248805 PMC10817257

[19] Shen TS Driscoll DA Islam W Bovonratwet P Haas SB Su EP. Modern internet search analytics and total joint arthroplasty: what are patients asking and reading online? J Arthroplasty. (2021) 36:1224–31. 10.1016/j.arth.2020.10.024 33162279 PMC7573653

[20] Sullivan B Platt B Joiner J Jacobs C Conley C Landy DC An investigation of google searches for knee osteoarthritis and stem cell therapy: what are patients searching online? HSS J. (2022) 18:485–9. 10.1177/15563316221089930 36263281 PMC9527551

[21] Yamaguchi S Kimura S Watanabe S Mikami Y Nakajima H Yamaguchi Y Internet search analysis on the treatment of rheumatoid arthritis: what do people ask and read online? PLoS One. (2023) 18:e0285869. 10.1371/journal.pone.0285869 37738275 PMC10516429

[22] Charnock D Shepperd S Needham G Gann R. DISCERN: an instrument for judging the quality of written consumer health information on treatment choices. J Epidemiol Community Health. (1999) 53:105–11. 10.1136/jech.53.2.105 10396471 PMC1756830

[23] OleanderSoftware. Readability studio 2019: professional edition (2019).

[24] Currie G Robbie S Tually P. ChatGPT and patient information in nuclear medicine: GPT-3.5 versus GPT-4. J Nucl Med Technol. (2023) 51:307–13. 10.2967/jnmt.123.266151 37699647

[25] King RC Samaan JS Yeo YH Mody B Lombardo DM Ghashghaei R. Appropriateness of ChatGPT in answering heart failure related questions. Heart Lung Circ. (2024) 33:123–30. 10.1016/j.hlc.2024.03.005 38821760

