# Supplementary material for: Enhancing patient education on the role of tibial osteotomy in the management of knee osteoarthritis using a customized ChatGPT: a readability and quality assessment
Source: Front Digit Health. 2025 Jan 3;6:1480381. doi: 10.3389/fdgth.2024.1480381 (PMC11738919; doi:10.3389/fdgth.2024.1480381)
Supplement: Supplementary file 1 [file Datasheet1.pdf]

Supplementary Table 1. Summary of Readability Formulae

Readability Test	Score Type	Description	Formula
**Flesch- Kincaid Reading Grade Level**	Grade Level	Designed for technical documents as part of the Kincaid Navy Personnel collection of tests. Applicable to broad array of disciplines.	G = (11.8 X (B/W)) + (0.39 X (W/S)) -15.59
**Flesch-Kincaid Reading Ease**	Index Score (0-100)	The standard test used by many US government agencies. Originally designed to newspaper readability. Best suited to school textbooks and technical documents. Scored from 0-100, with higher scores indicating easier readability.	I = (206.835 - (84.6 X (B/W)) - (1.015 X (W/S)))

G=Grade level; B= Number of syllables; W= Number of words; S= Number of sentences; RGL= Reading Grade Level; I= Flesch Index Score;
